# Timing of Surgical Intervention with Cochlear Implant in Patients with Large Vestibular Aqueduct Syndrome

**DOI:** 10.1371/journal.pone.0081568

**Published:** 2013-11-25

**Authors:** Hui-Chen Ko, Tien-Chen Liu, Li-Ang Lee, Wei-Chieh Chao, Yung-Ting Tsou, Shu-Hang Ng, Che-Ming Wu

**Affiliations:** 1 Department of Otolaryngology, Chang-Gung Memorial Hospital, College of Medicine, Chang-Gung University, Linkou, Taiwan; 2 Department of Otolaryngology, National Taiwan University Hospital, Taipei, Taiwan; 3 Molecular Imaging Center and Departments of Diagnostic Radiology and Medical Imaging, Chang-Gung University, Linkou, Taiwan; 4 Radiological Sciences, Chang-Gung University, Linkou, Taiwan; Baycrest Hospital, Canada

## Abstract

**Objectives:**

(1) To report the speech perception and intelligibility results of Mandarin-speaking patients with large vestibular aqueduct syndrome (LVAS) after cochlear implantation (CI); (2) to compare their performance with a group of CI users without LVAS; (3) to understand the effects of age at implantation and duration of implant use on the CI outcomes. The obtained data may be used to guide decisions about CI candidacy and surgical timing.

**Methods:**

Forty-two patients with LVAS participating in this study were divided into two groups: the early group received CI before 5 years of age and the late group after 5. Open-set speech perception tests (on Mandarin tones, words and sentences) were administered one year after implantation and at the most recent follow-up visit. Categories of auditory perception (CAP) and Speech Intelligibility Rating (SIR) scale scores were also obtained.

**Results:**

The patients with LVAS with more than 5 years of implant use (18 cases) achieved a mean score higher than 80% on the most recent speech perception tests and reached the highest level on the CAP/SIR scales. The early group developed speech perception and intelligibility steadily over time, while the late group had a rapid improvement during the first year after implantation. The two groups, regardless of their age at implantation, reached a similar performance level at the most recent follow-up visit.

**Conclusion:**

High levels of speech performance are reached after 5 years of implant use in patients with LVAS. These patients do not necessarily need to wait until their hearing thresholds are higher than 90 dB HL or PB word score lower than 40% to receive CI. They can do it “earlier” when their speech perception and/or speech intelligibility do not reach the performance level suggested in this study.

## Introduction

Large vestibular aqueduct syndrome (LVAS) was first discussed by Valvassori and Clemis in 1978 [1]. Children with LVAS may have unstable hearing (i.e., fluctuating or progressive hearing loss) during childhood. This progressively deteriorating hearing loss is usually associated with exercise, minor head trauma or upper respiratory tract infection [2]. 

Evidence from many previous studies suggests that cochlear implantation (CI) is effective in patients whose hearing condition or speech perception has worsened to a severe-to-profound level (i.e., they are unable to detect sounds quieter than 90 dB HL or score <40% on the monosyllabic word test) because of LVAS [3-10]. Miyamoto et al. [7] showed that their 14 adult patients attained a mean score of 69.2% on the sentence test after implantation but only achieved a mean score of 28% on the monosyllabic word test. The results of our previous research also indicated that after three years of CI use, patients with LVAS had better speech perception in the implanted ear than in the other ear with a hearing aid [10]. That finding indicates that although the implanted ear originally had poorer auditory and speech perception abilities, it improved considerably and surpassed the other ear over time.

Miyamoto et al. [7] demonstrated that 46% to 65% of patients in past studies were found to have progressive sensorineural hearing loss associated with LVAS. Gopen et al. [11] also mentioned that three long-term studies [12-14] reached similar conclusions. However, Emmett [15] and Zalza et al. [16] obtained contradictory results; they reported that most of their patients had stable hearing. Nevertheless, the follow-up spans of these studies were either short or ranged greatly. Regarding Mandarin-speaking patients with LVAS, Wu et al. [10] recruited 101 CI patients with LVAS and found that 75% of them had fluctuating hearing loss. Lai and Shiao [17] reported that most of their patients had stable hearing in at least one ear during follow-up; only 33% fluctuated. However, the sample size was rather small (twelve cases only).

Despite the effectiveness of cochlear implantation, clinicians are caught in a dilemma when dealing with the optimal timing of surgery for patients with LVAS. The dilemma is related to the hearing fluctuation. In one way, the hearing of these patients may fluctuate; although it may deteriorate to a profound level, it still can return to an aidable level. Therefore, implantation that is performed too early may not allow adequate time for hearing to recover on its own. On the other hand, waiting for too long may miss the best time frame to provide the secure auditory input that is required for normal speech and language development.

 As Table 1 shows, many studies have proposed that the best timing for implantation is when a patient’s hearing has deteriorated to a profound level [4,10,18,19]. In our previous study [10], we suggested that patients with LVAS should receive implantation within three months of when their hearing began to deteriorate and remain at the profound level. However, our clinical observations show that although many patients with LVAS have considerable residual hearing, some of them have high-frequency loss, which worsens their speech perception; many others have poor speech intelligibility. This implies that the level of residual hearing may not be an adequate indicator or criterion with which to determine implantation timing because it does not take speech perception and intelligibility into consideration. However, there are too few data available for clinicians and parents to evaluate how much improvement in speech perception and intelligibility can be expected after implantation. Only with information about these standards can the clinicians determine the best timing for implantation.

**Table 1:** Comparison among studies that addressed the timing of implantation and postsurgical speech perception performance.

Study	Number of patients	ImpAge (mean)	Level of deafness	Timing proposed	Outcome measures (mean)
Au & Gibson [4]	10	2.3-9.8 (6.7)	Profound	Sentence score < 40% with hearing aids or 3 significant decreases in hearing within 1 year	Word (43%); sentence (79%)
Miyamoto et al. [7]	9 children; 14 adults	9.9; 46.3	Profound	NA	Word (28%); sentence (69.2%)
Wu et al. [10]	12	1.8-7.3 (4.3)	Severe to profound	Within 3 months after hearing deteriorated and stayed at the profound level	Consonant (80%); tone (75%); word (86%); sentence (91.5-97%)^1^
Asma et al. [18]	10	2-22 (9.2)	Profound	Speech recognition < 40% at 70 dB	Word (69%); sentence (76%)
Chen et al. [19]	259	0.6-3	Profound	Age < 2	IT-MAIS (70.7)^2^

ImpAge: Age at implantation.

^1^ Medians are used to present the results.

^2^ The IT-MAIS score in this study was transformed into a percentage (i.e., total score/40*100).

Therefore, the present study aimed (1) to report the postoperative speech perception and intelligibility results of Mandarin-speaking patients with LVAS who received their implants before and after the age of 5 years (2), to examine the performance of patients who had used CIs for more than 5 years and compare them with a group of CI users without LVAS, and (3) to understand the effects of age at implantation and duration of implant use on the outcomes of cochlear implantation in patients with LVAS. Combining these three aspects may allow us to provide a reference for suggesting a surgical intervention timing. Our hypothesis is that if cochlear implantation can be considered based on not only hearing thresholds but also speech perception and intelligibility status, much better outcomes may be reached for this group of patients. 

## Materials and Methods

### Participants

Forty-two patients (22 boys and 20 girls) with LVAS participated in this study. Eighteen of them received CI before the age of five years (the early group), and 24 were implanted after the age of five years (the late group). As Table 2 shows, the early group received CI at a mean age of 3.3 ± 1.1 years. The mean duration of implant use was 6.9 ± 3.7 years. The late group was implanted at a mean age of 11.6 ± 8.4 years, and the mean duration of implant use was 3.8 ± 3.3 years. All of the participants had their CIs implanted between the years 2000 and 2012. 

**Table 2:** Background information and the categories of auditory perception and speech intelligibility rating results between the early and late groups.

	Early group (*n* = 18)			Late group (*n* = 24)			
	Mean ± SD	Median	Range	Mean ± SD	Median	Range	*P* value
Age at implantation	3.3 ± 1.1	3.1	1.8-4.9	10.3 ± 6.0	7.3	5.2-25.3	< 0.001*
Age at last test	10.5 ± 3.5	12.1	4.3-14.7	14.9 ± 7.0	12.3	7.6-30.3	0.079
Duration of Implant Use	7.3 ± 3.5	8.6	1.0-12.1	4.6 ± 3.3	2.9	1.3-12.1	0.015*
Categories of auditory perception							
Presurgical	2.4 ± 2.0	2	0-6	4.0 ± 2.0	4	1-7	0.016*
One year postsurgery	5.0 ± 1.1	5	3-7	5.5 ± 1.4	6	2-7	0.054
Most recent	6.2 ± 0.9	6	4-7	6.0 ± 1.2	6	3-7	0.406
Speech intelligibility rating							
Presurgical	1.9 ± 1.1	1.5	1-4	3.7 ± 1.3	4	1-5	< 0.001*
One year postsurgery	3.4 ± 1.1	3	2-5	4.2 ± 1.1	5	1-5	0.009*
Most recent	4.5 ± 0.9	5	2-5	4.3 ± 1.2	5	1-5	0.872

* A value of *P* < 0.05 using the 2-sided Mann-Whitney *U* test was considered significant.

SD: standard deviation.

Eighteen (43%) of the 42 subjects had used the implants for more than 5 years (LVAS group). For comparison of their performances with CI patients without LVAS, we further recruited 18 age- and gender-matched non-LVAS patients who underwent CI during the same period and also used the implants for at least five years (Table 3). All written informed consent forms signed by participants and guardians on the behalf of the minors/children participants involved in the present study were obtained before the test procedures took place. The study protocol and written informed consent form was approved by Chang-Gung Memorial Hospital Ethics Committee for Human Studies. 

**Table 3:** Background information and the categories of auditory perception and speech intelligibility rating results between the LVAS and non-LAVS groups.

	LVAS group (*n* = 18)			Non-LVAS group (*n* = 18)			
	Mean ± SD	Median	Range	Mean ± SD	Median	Range	*P* value
Age at implantation	5.4 ± 4.2	4.3	1.8-19.9	4.8 ± 3.0	4.3	1.7-13.3	0.800
Age at last test	14.4 ± 4.6	13.2	9.9-30.3	14.5 ± 2.8	13.5	11.4-21.3	0.359
Duration of Implant Use	9.0 ± 2.3	9.5	5.1-12.1	9.7 ± 1.5	10.3	6.5-12.5	0.343
Categories of auditory perception							
Presurgical	3.3 ± 1.8	4	1-6	2.1 ± 1.6	1	1-6	0.063
One year postsurgery	5.3 ± 0.9	5.5	4-7	4.6 ± 1.1	4	3-7	0.030*
Most recent (≥ 5 years after CI)	6.7 ± 0.5	7	6-7	6.4 ± 0.6	6	5-7	0.209
Speech intelligibility rating							
Presurgical	2.5 ± 1.3	2.5	1-4	1.7 ± 1.1	1	1-5	0.039*
One year postsurgery	3.8 ± 1.2	4	2-5	3.1 ± 1.0	3	2-5	0.044*
Most recent (≥ 5 years after CI)	4.8 ± 0.4	5	4-5	4.7 ± 0.5	5	4-5	0.214

* A value of *P* < 0.05 using the 2-sided Mann-Whitney *U* test was considered significant.

SD: standard deviation. CI: cochlear implantation.

### Test Materials

#### Speech perception tests

Four open-set speech perception tests were used to examine the patients with LVAS, including an easy-sentence test, a difficult-sentence test, a phonetically balanced (PB) word recognition test and a Mandarin tone recognition test. 

The easy-sentence test was developed by Lin et al. (unpublished materials) and based on the Central Institute for the Deaf (CID) Everyday Sentence test [20]. It included 15 sentences varying in length from two to ten words. Each sentence contained one to seven key words chosen from a corpus of words that are familiar to the subjects in their daily communication, for example, “book” and “car.” The difficult-sentence test consisted of 20 sentences varying in length from two to twelve words. Each sentence embedded one to ten key words that were to be scored, but these key words were less familiar to children, such as “examine” and “dormitory.” The PB word recognition test, developed by Wang and Su [21], included 25 monosyllabic words. The 80 monosyllabic Mandarin words for the Mandarin tone recognition test were developed by Liu et al. [22]. The four Mandarin tones (flat, rising, dipping, falling) were equally distributed throughout the word list. There were a total of four lists of easy sentences and tones, three lists of difficult sentences and five lists of PB words. The materials used for each test are shown in Appendix S1. 

#### Categories of Auditory Perception (CAP) and Speech Intelligibility Rating (SIR) scales

Other than speech perception, the auditory receptive abilities and speech intelligibility of these children were rated using the CAP and the SIR scales, respectively. The CAP is an 8-point nonlinear and hierarchical rating scale. Its scores range from the lowest level (0) of being unaware of environmental sounds to the highest level (7) of having the ability to converse on the telephone with a familiar person (see Table 4). Its reliability has been proven [23]. The SIR is a 5-point nonlinear scale that reflects children’s speech production intelligibility from the lowest level (1) of being unintelligible to the highest level (5) of being easily understood by all listeners (see Table 5). The reliability of the scale has been confirmed [24,25]. 

**Table 4:** Categorical Auditory Performance (CAP) criteria.

Rating	Criterion
7	Uses the telephone with a known listener
6	Understands conversation without lip-reading
5	Understands common phrases without lip-reading
4	Discriminates some speech sounds without lip-reading
3	Identifies environmental sounds
2	Responds to speech sounds
1	Is aware of environmental sounds
0	Has no awareness of environmental sounds

**Table 5:** Speech Intelligibility Rating Scale (SIR) criteria.

Rating	Criterion
5	Connected speech is intelligible to all listeners. Child is understood easily in everyday contexts.
4	Connected speech is intelligible to a listener who has some experience with deaf people’s speech
3	Connected speech is intelligible to a listener who concentrates and lip-reads
2	Connected speech is unintelligible. Intelligible speech is developing for single words when context and lip-reading cues are available
1	Connected speech is unintelligible. Spoken words are pre-recognizable; the primary mode of communication may be manual.

### Test procedures

This study took place at the CI center of the Department of Otolaryngology, Chang-Gung Memorial Hospital, a tertiary referral hospital in Taiwan. The CAP and SIR scales were rated either by the parents or by the speech-language therapists who knew the children best. The speech perception tests were conducted in a sound-insulated booth. The stimulus level was controlled at 60 dB HL. The children were asked to orally repeat the words or sentences they heard. The easy-sentence and difficult-sentence tests were scored based on how many key words the child correctly repeated, while the PB word test was scored according to how many words the child correctly repeated. The Mandarin tone recognition test was scored according to the tones only. A word would be counted correct as long as its tone was correctly repeated; the mistakes the patient made on the vowel or consonant were overlooked. The number of the correctly repeated items for each test was converted into percentages (% correct) for further analysis. To minimize learning effects, the testing lists were randomly selected. The answers were recorded for later evaluation, and the same procedure was administered preoperatively and postoperatively. The following analysis was primarily based on the children’s scores before implantation, one year after implantation and most recently.

### Statistical analysis

Descriptive statistics were summarized using frequencies, percentages, median, means, standard deviations (SDs) and ranges. A Mann-Whitney U test for independent groups was used for between-group comparisons of test results. A Wilcoxon signed-rank test was used for dependent-group comparisons of test results. Categorical variables were analyzed using the Fisher’s exact test. A Spearman nonparametric correlation test was used to investigate the relationships of the scores of outcome measures, age at implantation and duration of implant use. A value of P < 0.05 was considered significant. Statistical analyses were conducted using SPSS software (version 17.0; SPSS; SPSS, Inc., Chicago, IL, USA).

## Results

### Comparison between the early group and the late group

Before implantation, the early group had a median CAP score of 2 and a median SIR score of 1.5, while the late group obtained a median of 5 and 4 for the CAP and the SIR, respectively (Table 2). Although the late group seemed to have some degree of intelligible speech, more than one third of the patients in this group had an SIR score below 3 (Table 6). Nevertheless, the early group had a significantly higher proportion of SIR score < 3 than the late group at baseline (89% vs. 38%, P < 0.001) and one year postoperatively (56% vs. 21%, P = 0.027). Interestingly, the early group patients overtook the late group patients at their most recent follow-up (11% vs. 21%, P = 0.679).

**Table 6:** Number of patients with a SIR score below 3 for evaluations made before implantation, one year after implantation and at the most recent follow-up visit.

	Early group (*n* = 18)	Late group (*n* = 24)	*P* value
Presurgery			
SIR < 3	16 (89%)	9 (38%)	0.001
SIR ≥ 3	2 (11%)	15 (62%)	
One year postsurgery			
SIR < 3	10 (56%)	5 (21%)	0.027
SIR ≥ 3	8 (44%)	19 (79%)	
Most recently			
SIR < 3	2 (11%)	5 (21%)	0.679
SIR ≥ 3	16 (89%)	19 (79%)	

Note: Values are number of case (%).

*A value of *P* < 0.05 using the 2-sided Fisher’s exact test was considered significant.

SIR: speech intelligibility rating score.

After one year of implant use, the late group reached a median CAP score of 6 and a median SIR score of 5. Their scores were higher than those of the early group, with a median CAP score of 5 and a median SIR score of 3 (Table 2). The Mann-Whitney U test showed that the SIR score differed significantly between the two groups, while the CAP score did not (see Table 2). The late group also performed better on three of the speech perception tests (the tone recognition test, the easy sentence test and the difficult sentence test) than the early group did (Table 7). The tone recognition score of the late group (67.3 ± 19.1%) was significantly higher than that of the early group (48.1 ± 26.1%, U = 105.5, Z = -2.109, P = 0.020). The between-group differences for the other three speech perception tests did not reach significance (P > 0.05).

**Table 7:** Comparison of speech perception test scores obtained one year after implantation and most recently between the early and late groups.

	Early group (*n* = 18)		Late group (*n* = 24)		
% correct	Mean ± SD	Range	Mean ± SD	Range	*P* value
One year postsurgery					
Tone	48.1 ± 26.1	0-80	67.3 ± 19.1	30-100	0.020*
Sentence 1	76.3 ± 29.1	25-100	80.6 ± 25.3	12-100	0.665
Sentence 2	70.0 ± 34.7	18-95	80.0 ± 23.8	27-100	0.500
PB word	82.9 ± 7.6	72-6	80.3 ± 15.1	40-96	0.635
Most recently					
Tone	67.2 ± 32.5	0-100	76.8 ± 15.2	30-100	0.261
Sentence 1	92.6 ± 16.6	36-100	84.8 ± 25.4	12-100	0.242
Sentence 2	87.9 ± 21.7	18-100	83.9 ± 22.6	27-100	0.575
PB word	86.7 ± 13.3	64-100	81.7 ± 13.3	56-100	0.274

* A value of *P* < 0.05 using the 2-sided Mann-Whitney *U* test was considered significant.

SD: standard deviation. Sentence 1: Easy sentence. Sentence 2: Difficult sentence. PB: phonetically balanced.

 At the most recent evaluation, the early group showed improvements in their CAP and SIR scores, while the late group remained at the same level they had achieved one year postsurgery (Table 2). The CAP and SIR scores of the two groups did not differ significantly. Regarding the speech perception tests, the early group also showed obvious improvement on all four of the tests (Table 7). A Wilcoxon sgned-rank test indicated that the tone recognition and easy-sentence scores that the early group obtained at the latest evaluation were significantly higher than the scores it obtained during the first year after implantation (Z = -2.288, P = 0.022 for tone recognition; Z = -2.371, P = 0.018 for easy sentences). Moreover, the early group outperformed the late group on the two sentence tests and the PB word test, although the differences did not reach significance.

### Comparison between the LVAS group and the non-LVAS group

**Figure 1:**
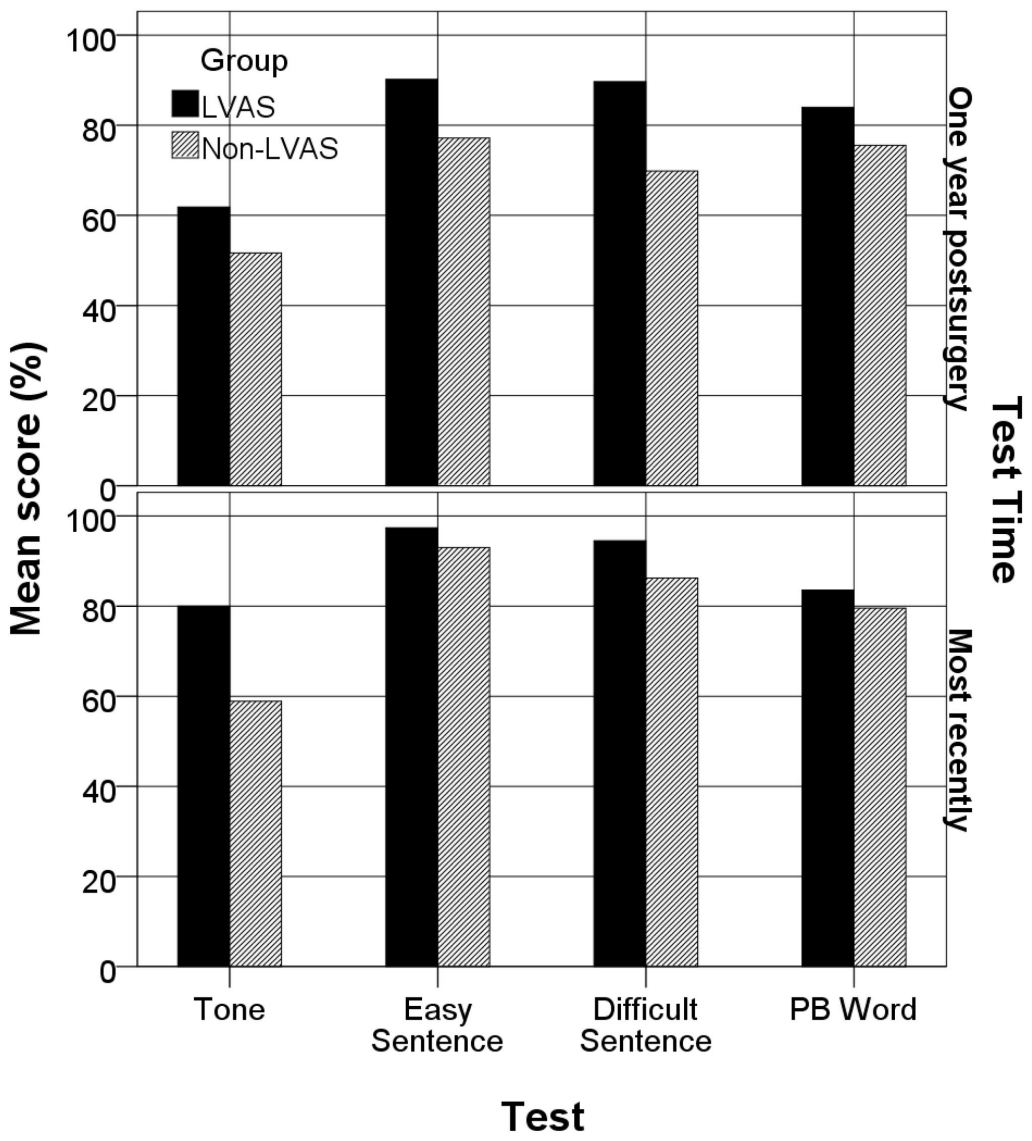
Mean scores on speech perception tests for the LVAS group and Non-LVAS group. The mean scores for four speech perception tests obtained one year after implantation and at the most recent follow-up visit by the LVAS group and the non-LVAS group.

As Table 8 shows, after 5 years of implant use, the LVAS group achieved a mean score higher than 80% on all four speech perception tests, and its median scores were all above 85%, meaning that half of the patients in this group could score higher than 85% after using CIs for more than 5 years. The LVAS group also exhibited better speech perception than the non-LVAS group did on the evaluations conducted one year after implantation and most recently (Figure 1). A Mann-Whitney U test indicated that the tone recognition score obtained most recently by the LVAS group was statistically higher than that of the non-LVAS group (U = 76.0, Z = -2.750, P = 0.006). The significance level was not reached for the other three tests. Similarly, the LVAS group had higher CAP and SIR scores than the non-LVAS group did on the evaluations conducted preoperatively, one year after implantation and most recently (Table 3). The Mann-Whitney U test showed that the CAP and SIR scores the LVAS group obtained one year after implantation were significantly higher than those of the non-LVAS group (U = 90.0, Z = -2.165, P = .030 for CAP; U = 94.0, Z = -2.015, P = .044 for SIR).

**Table 8:** Comparison of speech perception test scores obtained by patients more than five years after implantation and most recently between the LVAS and non-LVAS groups.

	LVAS (*n* = 18)			Non-LVAS group (*n* = 18)			
% correct	Mean ± SD	Median	Range	Mean ± SD	Median	Range	*P* value
One year postsurgery							
Tone	60.7 ± 22.8	70	20-100	51.7 ± 23.3	50	20-90	0.197
Sentence 1	90.2 ± 15.3	95	48-100	77.2 ± 25.0	89	38-100	0.404
Sentence 2	89.7 ± 8.3	90.5	77-98	69.8 ± 26.9	78.5	23-100	0.173
PB word	84.0 ± 4.0	84	80-88	75.6 ± 14.1	76	60-100	0.261
Most recently							
Tone	80.0 ± 22.0	90	10-100	58.9 ± 24.2	60	20-100	0.006*
Sentence 1	97.4 ± 6.0	100	76-100	93.0 ± 12.3	97	52-100	0.093
Sentence 2	94.5 ± 8.4	98	70-100	86.2 ± 15.6	90	45-100	0.079
PB word	85.6 ± 10.3	88	64-100	79.6 ± 19.9	86	40-100	0.836

* A value of *P* < 0.05 using the 2-sided Mann-Whitney *U* test was considered significant.

SD: standard deviation. Sentence 1: Easy sentence. Sentence 2: Difficult sentence. PB: phonetically balanced.

### The effects of age at implantation and duration of implant use

Using the Spearmen's correlation test, we found that age at implantation was significantly associated with presurgical CAP (r = 0.450, P = 0.004) and presurgical SIR (r = 0.635, P < 0.001), one-year postsurgical SIR (r = 0.464, P = 0.004) and tone recognition (r = 0.421, P = 0.009), and most recent easy sentence (r = -0.343, P = 0.028) in patients with LVAS. No significant correlation was found between age at implantation and the most recent CAP/SIR scores.

 Moreover, duration of implant use was significantly correlated to presurgical CAP (r = -0.343, P = 0.030) and most recent CAP (r = 0.614, P < 0.001), SIR (r = 0.420, P = 0.006), tone recognition (r = 0.363, P = 0.021), easy-sentence score (r = 0.467, P = 0.002), and difficult-sentence score (r = 0.376, P = 0.018).

## Discussion

The speech perception performances of CI users with LVAS have been investigated by many studies in different languages, yet this study is the first to report the postoperative speech perception and intelligibility results in a cohort of Mandarin-speaking CI patients with LVAS for the purpose of providing a reference for suggesting a timing of surgical intervention. 

To better elucidate our results, we divided these patients into groups according to age at implantation because implantation age is often reported to have an effect on the development of speech perception and intelligibility [19,26,27]. However, the results of the present study show that younger age at implantation is not necessarily related to better speech perception and intelligibility outcomes. Rather, the late group generally had better speech perception performance and higher CAP/SIR scores compared with the early group after one year of implant use, indicating the late group’s rapid improvements after cochlear implantation. This probably resulted from the late group’s preoperative residual hearing and language experience. After some years of implant use, the early group showed significant improvement at their most recent follow-up and reached a similar performance level as the late group did in the subjective and objective evaluations. This finding shows that earlier implantation allows patients with LVAS to develop their language skills through a gradual process similar to that undergone by normal-hearing children. Yet, it does not necessarily lead to better speech perception outcomes because those who get implanted at a later age can take advantage of their past language experience to achieve good speech performance after their fluctuating hearing is restored and stabilized by CI.

Many of the patients in the late group received CI because their unstable hearing continued to fluctuate and to deteriorate, leading to unsatisfactory speech intelligibility despite considerable residual hearing and many years of hearing aid use. One factor worth noticing is that more than half of the participants were implanted after the age of 5 years, suggesting that many patients with LVAS undergo cochlear implantation at a later age, possibly because of the fluctuating hearing loss. Many previous studies also used a large proportion of postlingually deafened participants [7,17,28-31], and studies that focused on prelingual implantees were relatively unavailable [10,19,32,33]. These studies showed that many adult patients with LVAS need cochlear implantation despite many years of experience with hearing aids. We thus suggest that if patients have unstable hearing, high-resolution computed tomography and genetic examinations should take place to confirm the cause of hearing fluctuation, and regular follow-up is needed.

The evaluations, however, should not solely depend on patients’ auditory performances and PB word scores (which are usually used to define hearing loss level) because LVAS patients have unstable/fluctuating hearing. The CAP scores only reflect the patients’ auditory level at the time of the evaluation and fail to indicate whether the patient has unstable hearing. Because of unstable hearing, auditory performance and hearing thresholds are not entirely reliable. The PB word test also reveals that LVAS patients progress slowly on the test after having used the implants for one year. These outcomes suggest that speech perception and speech intelligibility are better evaluation tools because they represent a combination of total past hearing experience and language acquisition results before implantation. Thus, speech intelligibility (SIR scale) and a more comprehensive speech perception test should be administered during preoperative and postoperative evaluations.

The observation that the long-term effectiveness of CI was better than that of the hearing aids suggests that the inclusion criteria for CI candidacy could be expanded [34]. Currently, it remains unclear whether and when the patient with fluctuating or progressive hearing loss should receive implants. The selection of CI candidates primarily focuses on patients with severe-to-profound hearing loss defined by their hearing threshold or PB scores. Patients with unstable hearing could thus be easily overlooked.

Due to unstable hearing, some patients or parents of the patients hesitate over the decision of CI. They fear that CI may destroy the residual hearing, affecting their current speech perception level, or rule out the possibility that the hearing could recover on its own. Many of them thus delay the implantation surgery. This delay in implantation may cause the patients to miss the optimal time frame for developing good speech intelligibility and proper learning skills because the unstable hearing condition remains unchanged, which may further affect their life quality [27]. In view of such cases, we wish to establish a reference for evaluating cochlear implantation candidacy and suggesting an optimal surgical timing. Our results suggest that patients with LVAS have the potential to score higher than 80% on speech perception tests and to reach the highest level of speech intelligibility after using implants for more than 5 years. Those who get implanted after 5 years of age even achieve it within one year after implantation. Therefore, although we cannot suggest a specific time frame for surgical intervention based on our data, we encourage patients with LVAS to consider cochlear implantation if they are not able to reach the performance level suggested in this study and do not show much improvement for more than three months. It may not be necessary to wait until they can only hear sounds louder than 90 dB HL or score below 40% on a PB word test (i.e., until they are defined as having profound hearing loss). For further studies, we suggest recruiting more subjects from different clinics to do a larger-scale long-term outcome survey, which may help strengthen our proposal.

## Conclusion

In this study, we demonstrate that the postoperative speech perception and intelligibility are satisfactory after more than 5 years of CI use in a small cohort of children with LVAS. Although our preliminary results are promising, further research such as a large-scale long-term outcome survey is indicated. Nevertheless, we suggest LVAS patients with insufficient speech perception or speech intelligibility performance to undergo earlier CI because it may provide a better opportunity and greater amount of time to develop their language skills in a manner similar to that of normal-hearing children. 

## Supporting Information

Appendix S1 **a. Easy sentence list for the speech perception test (English translation)**. The key words are underlined. **b. Difficult sentence list for the speech perception test (English translation)**. The key words are underlined. **c. Monosyllabic PB word list for the speech perception test**. Pinyin characters are used. English translations are parenthesized. **d. Word list for the Mandarin tone recognition test**. Pinyin characters are used. English translations are parenthesized.
(DOC)
