# Supplementary material for: Timing of Surgical Intervention with Cochlear Implant in Patients with Large Vestibular Aqueduct Syndrome
Source: PLoS One. 2013 Nov 25;8(11):e81568. doi: 10.1371/journal.pone.0081568 (PMC3839901; doi:10.1371/journal.pone.0081568)
Supplement: Appendix S1 — a. Easy sentence list for the speech perception test (English translation). The key words are underlined. b. Difficult sentence list for the speech perception test (English translation). The key words are underlined. c. Monosyllabic PB word list for the speech perception test. Pinyin characters are used. English translations are parenthesized. d. Word list for the Mandarin tone recognition test. Pinyin characters are used. English translations are parenthesized. (DOC) [file pone.0081568.s001.doc]

**Appendices**

**Appendix S1a.** Easy sentence list for the speech perception test (English translation). The key words are underlined.

(1) This book is good.
(2) Have you brought all your stuff?
(3) Be careful when driving the car.
(4) It does not matter.
(5) Turn off the television.
(6) Where is the newspaper?
(7) Excuse me, is the boss there?
(8) That coat looks really good.
(9) I am having a headache.
(10) Tomorrow is a holiday. Do you want to go out?
(11) It seems that I am having a fever.
(12) Please don’t smoke.
(13) The weather is quite nice today.
(14) How many brothers and sisters do you have?
(15) How many people are there in your family?

**Appendix S1b.** Difficult sentence list for the speech perception test (English translation). The key words are underlined.

(1) Can you hear what he is saying?
(2) How is your health condition?
(3) How about it?
(4) We will go there right after having dinner.
(5) Who is in there?
(6) Very busy.
(7) This movie is very good.
(8) We often go to the park.
(9) This building is taller than that one.
(10) The doctor comes to carry out examinations on him everyday.
(11) Many books are put on the table.
(12) I am so tired.
(13) Is sister busily working?
(14) I am a student.
(15) That novel is very interesting.
(16) There will be a movie today evening.
(17) He has graduated for about two years.
(18) When he is anxious, he cannot even speak a word.
(19) Sit down, please!
(20) It is summer now.

**Appendix S1c.** Monosyllabic PB word list for the speech perception test. Pinyin characters are used. English translations are parenthesized.

| bà (dad) | zhǐ (only) | nǔ (effort) | ling (bell) | qì (air) |
| --- | --- | --- | --- | --- |
| nioú (cow) | zuǒ (left) | jìn (nearby) | yén (salt) | gòu (enough) |
| jiěn (minus) | fù (father) | kāi (open) | zhú (bamboo) | rù (enter) |
| jiǎng (talk) | cīng (star) | lí (pear) | zhè (this) | zhàn (battle) |
| yā (duck) | yào (want) | yú (fish) | jiěh (sister) | guó (nation) |

**Appendix S1d.** Word list for the Mandarin tone recognition test. Pinyin characters are used. English translations are parenthesized.

| Tone 1 | Tone 2 | Tone 3 | Tone 4 |
| --- | --- | --- | --- |
| bi (force) | bi (nose) | bi (compare) | bi (close) |
| di (low) | di (enemy) | di (bottom) | di (ground) |
| shu (book) | shu (uncle) | shu (rat) | shu (tree) |
| tu (protrude) | tu (picture) | tu (soil) | tu (rabbit) |
| ma (mother) | ma (sesame) | ma (horse) | ma (blame) |
| ba (eight) | ba (uproot) | ba (grip) | ba (dad) |
| po (slope) | po (old woman) | po (considerably) | po (broken) |
| ke (trunk) | ke (shell) | ke (permit) | ke (class) |
| xie (some) | xie (shoe) | xie (write) | xie (thank) |
| pai (pat) | pai (row) | bai (put) | bai (worship) |
| bei (cup) | pei (accompany) | bei (north) | bei (blanket) |
| bao (bag) | bao (thin) | bao (baby) | bao (hug) |
| mao (cat) | mao (fur) | hao (good) | mao (hat) |
| tou (steal) | tou (head) | dou (steep) | dou (bean) |
| ban (squad) | pan (plate) | ban (board) | ban (half) |
| dan (single) | tan (talk about) | dan (gall) | dan (egg) |
| ben (run quickly) | pen (basin) | ben (origin) | ben (stupid) |
| tang (soup) | tang (sugar) | tang (lie down) | tang (hot) |
| bang (help) | pang (side) | bang (tie together) | bang (excellent) |
| deng (lamp) | teng (pain) | deng (wait) | deng (stool) |
